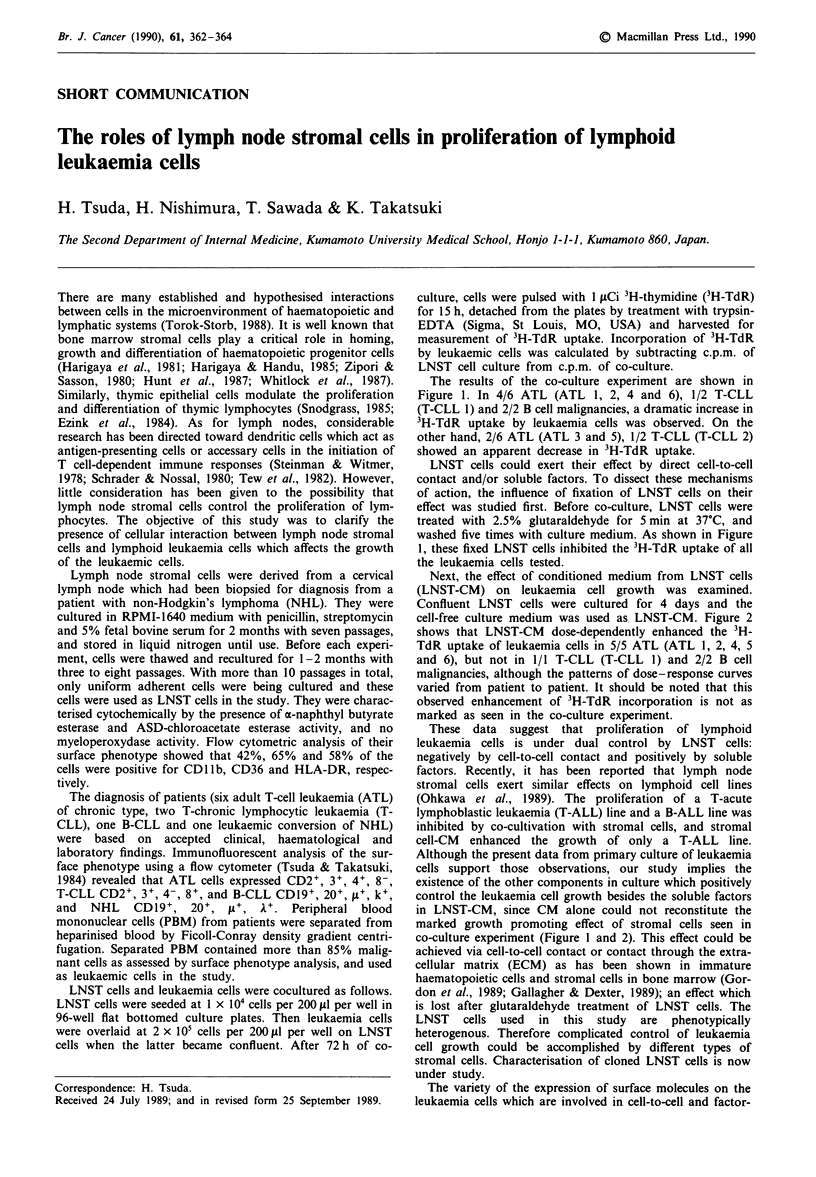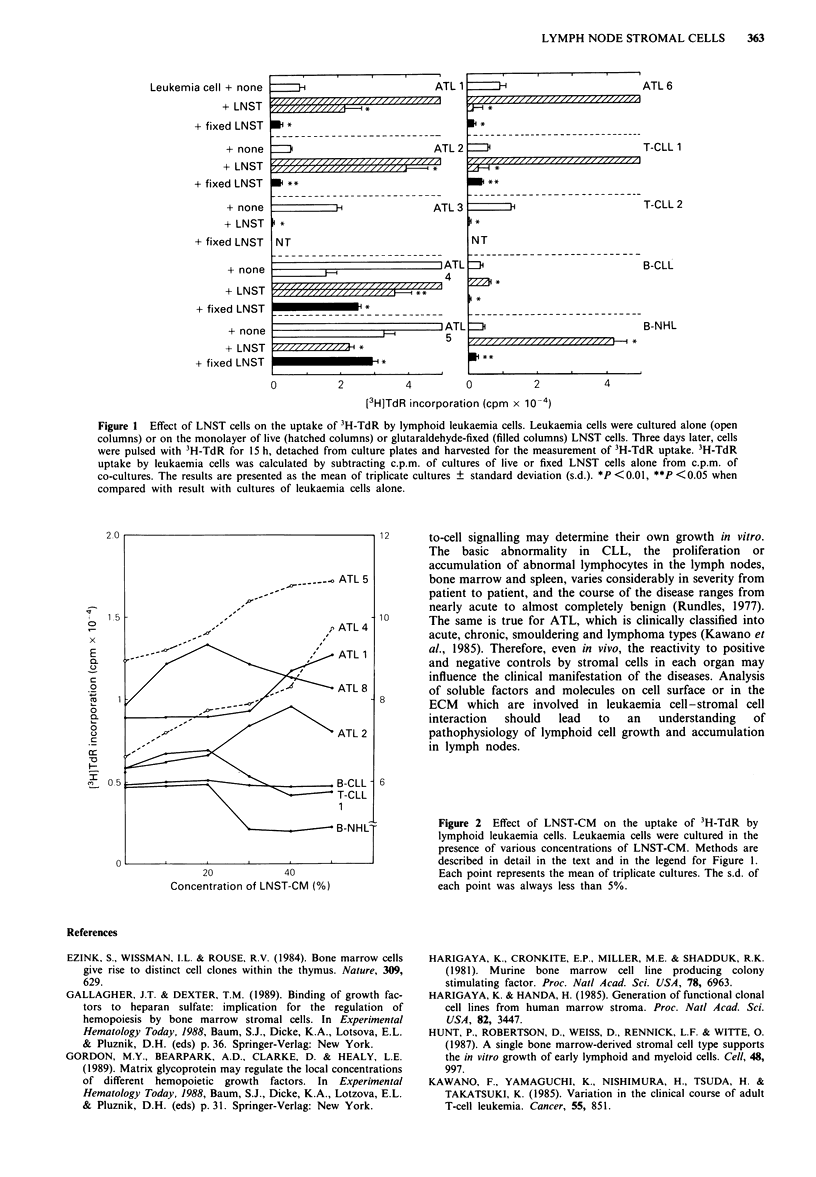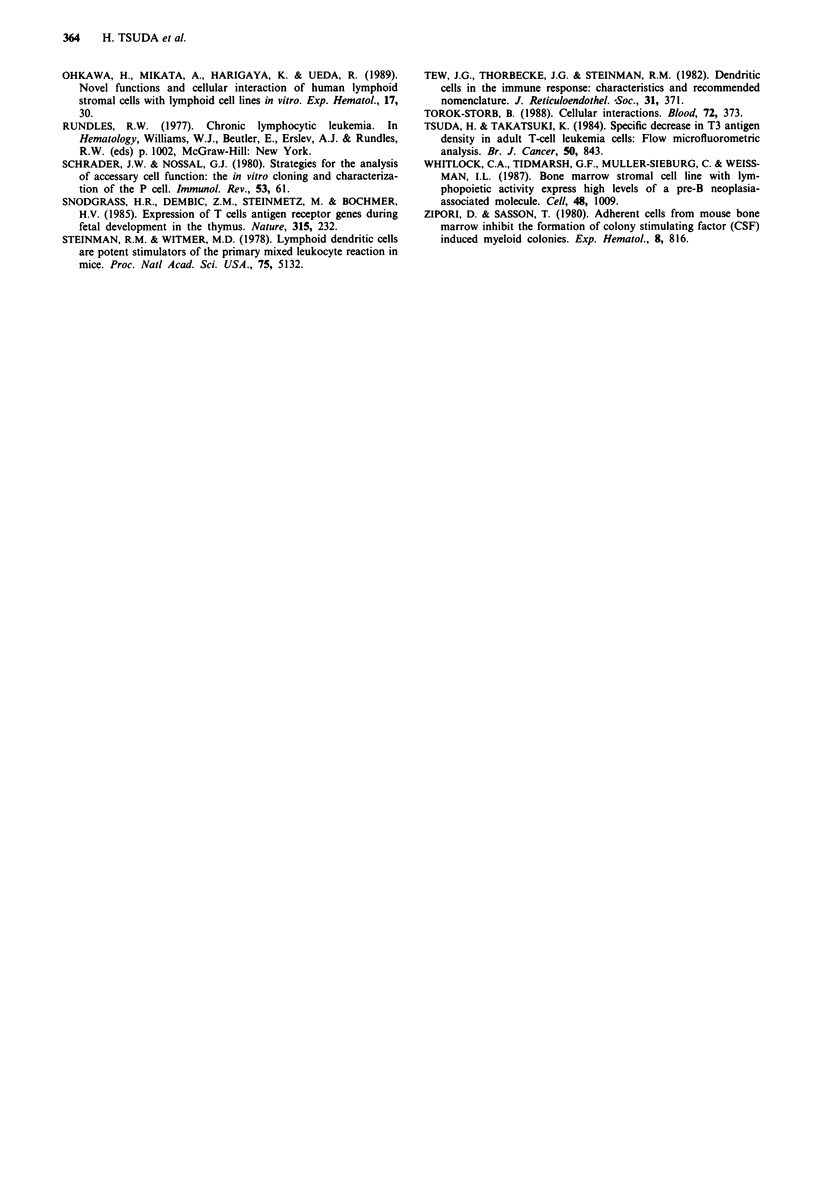# The roles of lymph node stromal cells in proliferation of lymphoid leukaemia cells.

**DOI:** 10.1038/bjc.1990.79

**Published:** 1990-03

**Authors:** H. Tsuda, H. Nishimura, T. Sawada, K. Takatsuki

**Affiliations:** Second Department of Internal Medicine, Kumamoto University Medical School, Japan.


Br. J. Cancer (1990), 61, 362-364 © Macmillan Press Ltd., 1990

SHORT COMMUNICATION

### The roles of lymph node stromal cells in proliferation of lymphoid leukaemia cells

H. Tsuda, H. Nishimura, T. Sawada & K. Takatsuki

*The Second Department of Internal Medicine, Kumamoto University Medical School, Honjo 1-1-1, Kumamoto 860, Japan.*

There are many established and hypothesised interactions between cells in the microenvironment of haematopoietic and lymphatic systems (Torok-Storb, 1988). It is well known that bone marrow stromal cells play a critical role in homing, growth and differentiation of haematopoietic progenitor cells (Harigaya *et al.*, 1981; Harigaya & Handu, 1985; Zipori & Sasson, 1980; Hunt *et al.*, 1987; Whitlock *et al.*, 1987). Similarly, thymic epithelial cells modulate the proliferation and differentiation of thymic lymphocytes (Snodgrass, 1985; Ezink *et al.*, 1984). As for lymph nodes, considerable research has been directed toward dendritic cells which act as antigen-presenting cells or accessary cells in the initiation of T cell-dependent immune responses (Steinman & Witmer, 1978; Schrader & Nossal, 1980; Tew *et al.*, 1982). However, little consideration has been given to the possibility that lymph node stromal cells control the proliferation of lymphocytes. The objective of this study was to clarify the presence of cellular interaction between lymph node stromal cells and lymphoid leukaemia cells which affects the growth of the leukaemic cells.

Lymph node stromal cells were derived from a cervical lymph node which had been biopsied for diagnosis from a patient with non-Hodgkin's lymphoma (NHL). They were cultured in RPMI-1640 medium with penicillin, streptomycin and 5% fetal bovine serum for 2 months with seven passages, and stored in liquid nitrogen until use. Before each experiment, cells were thawed and recultured for 1-2 months with three to eight passages. With more than 10 passages in total, only uniform adherent cells were being cultured and these cells were used as LNST cells in the study. They were characterised cytochemically by the presence of α-naphthyl butyrate esterase and ASD-chloroacetate esterase activity, and no myeloperoxydase activity. Flow cytometric analysis of their surface phenotype showed that 42%, 65% and 58% of the cells were positive for CD11b, CD36 and HLA-DR, respectively.

The diagnosis of patients (six adult T-cell leukaemia (ATL) of chronic type, two T-chronic lymphocytic leukaemia (T-CLL), one B-CLL and one leukaemic conversion of NHL) were based on accepted clinical, haematological and laboratory findings. Immunofluorescent analysis of the surface phenotype using a flow cytometer (Tsuda & Takatsuki, 1984) revealed that ATL cells expressed $CD2^+$, $3^+$, $4^+$, $8^-$, T-CLL $CD2^+$, $3^+$, $4^-$, $8^+$, and B-CLL $CD19^+$, $20^+$, $\mu^+$, $k^+$, and NHL $CD19^+$, $20^+$, $\mu^+$, $\lambda^+$. Peripheral blood mononuclear cells (PBM) from patients were separated from heparinised blood by Ficoll-Conray density gradient centrifugation. Separated PBM contained more than 85% malignant cells as assessed by surface phenotype analysis, and used as leukaemic cells in the study.

LNST cells and leukaemia cells were cocultured as follows. LNST cells were seeded at $1 \times 10^4$ cells per 200 μl per well in 96-well flat bottomed culture plates. Then leukaemia cells were overlaid at $2 \times 10^5$ cells per 200 μl per well on LNST cells when the latter became confluent. After 72 h of co-culture, cells were pulsed with 1 μCi $^3$H-thymidine ($^3$H-TdR) for 15 h, detached from the plates by treatment with trypsin-EDTA (Sigma, St Louis, MO, USA) and harvested for measurement of $^3$H-TdR uptake. Incorporation of $^3$H-TdR by leukaemic cells was calculated by subtracting c.p.m. of LNST cell culture from c.p.m. of co-culture.

The results of the co-culture experiment are shown in Figure 1. In 4/6 ATL (ATL 1, 2, 4 and 6), 1/2 T-CLL (T-CLL 1) and 2/2 B cell malignancies, a dramatic increase in $^3$H-TdR uptake by leukaemia cells was observed. On the other hand, 2/6 ATL (ATL 3 and 5), 1/2 T-CLL (T-CLL 2) showed an apparent decrease in $^3$H-TdR uptake.

LNST cells could exert their effect by direct cell-to-cell contact and/or soluble factors. To dissect these mechanisms of action, the influence of fixation of LNST cells on their effect was studied first. Before co-culture, LNST cells were treated with 2.5% glutaraldehyde for 5 min at 37°C, and washed five times with culture medium. As shown in Figure 1, these fixed LNST cells inhibited the $^3$H-TdR uptake of all the leukaemia cells tested.

Next, the effect of conditioned medium from LNST cells (LNST-CM) on leukaemia cell growth was examined. Confluent LNST cells were cultured for 4 days and the cell-free culture medium was used as LNST-CM. Figure 2 shows that LNST-CM dose-dependently enhanced the $^3$H-TdR uptake of leukaemia cells in 5/5 ATL (ATL 1, 2, 4, 5 and 6), but not in 1/1 T-CLL (T-CLL 1) and 2/2 B cell malignancies, although the patterns of dose-response curves varied from patient to patient. It should be noted that this observed enhancement of $^3$H-TdR incorporation is not as marked as seen in the co-culture experiment.

These data suggest that proliferation of lymphoid leukaemia cells is under dual control by LNST cells: negatively by cell-to-cell contact and positively by soluble factors. Recently, it has been reported that lymph node stromal cells exert similar effects on lymphoid cell lines (Ohkawa *et al.*, 1989). The proliferation of a T-acute lymphoblastic leukaemia (T-ALL) line and a B-ALL line was inhibited by co-cultivation with stromal cells, and stromal cell-CM enhanced the growth of only a T-ALL line. Although the present data from primary culture of leukaemia cells support those observations, our study implies the existence of the other components in culture which positively control the leukaemia cell growth besides the soluble factors in LNST-CM, since CM alone could not reconstitute the marked growth promoting effect of stromal cells seen in co-culture experiment (Figure 1 and 2). This effect could be achieved via cell-to-cell contact or contact through the extracellular matrix (ECM) as has been shown in immature haematopoietic cells and stromal cells in bone marrow (Gordon *et al.*, 1989; Gallagher & Dexter, 1989); an effect which is lost after glutaraldehyde treatment of LNST cells. The LNST cells used in this study are phenotypically heterogenous. Therefore complicated control of leukaemia cell growth could be accomplished by different types of stromal cells. Characterisation of cloned LNST cells is now under study.

The variety of the expression of surface molecules on the leukaemia cells which are involved in cell-to-cell and factor-

Correspondence: H. Tsuda.
Received 24 July 1989; and in revised form 25 September 1989.

**Figure 1** Effect of LNST cells on the uptake of $^3$H-TdR by lymphoid leukaemia cells. Leukaemia cells were cultured alone (open columns) or on the monolayer of live (hatched columns) or glutaraldehyde-fixed (filled columns) LNST cells. Three days later, cells were pulsed with $^3$H-TdR for 15 h, detached from culture plates and harvested for the measurement of $^3$H-TdR uptake. $^3$H-TdR uptake by leukaemia cells was calculated by subtracting c.p.m. of cultures of live or fixed LNST cells alone from c.p.m. of co-cultures. The results are presented as the mean of triplicate cultures ± standard deviation (s.d.). *$P < 0.01$, **$P < 0.05$ when compared with result with cultures of leukaemia cells alone.

**Figure 2** Effect of LNST-CM on the uptake of $^3$H-TdR by lymphoid leukaemia cells. Leukaemia cells were cultured in the presence of various concentrations of LNST-CM. Methods are described in detail in the text and in the legend for Figure 1. Each point represents the mean of triplicate cultures. The s.d. of each point was always less than 5%.

to-cell signalling may determine their own growth *in vitro*. The basic abnormality in CLL, the proliferation or accumulation of abnormal lymphocytes in the lymph nodes, bone marrow and spleen, varies considerably in severity from patient to patient, and the course of the disease ranges from nearly acute to almost completely benign (Rundles, 1977). The same is true for ATL, which is clinically classified into acute, chronic, smouldering and lymphoma types (Kawano *et al.*, 1985). Therefore, even *in vivo*, the reactivity to positive and negative controls by stromal cells in each organ may influence the clinical manifestation of the diseases. Analysis of soluble factors and molecules on cell surface or in the ECM which are involved in leukaemia cell–stromal cell interaction should lead to an understanding of pathophysiology of lymphoid cell growth and accumulation in lymph nodes.

### References

EZINK, S., WISSMAN, I.L. & ROUSE, R.V. (1984). Bone marrow cells give rise to distinct cell clones within the thymus. *Nature*, **309**, 629.

GALLAGHER, J.T. & DEXTER, T.M. (1989). Binding of growth factors to heparan sulfate: implication for the regulation of hemopoiesis by bone marrow stromal cells. In *Experimental Hematology Today, 1988*, Baum, S.J., Dicke, K.A., Lotsova, E.L. & Pluznik, D.H. (eds) p. 36. Springer-Verlag: New York.

GORDON, M.Y., BEARPARK, A.D., CLARKE, D. & HEALY, L.E. (1989). Matrix glycoprotein may regulate the local concentrations of different hemopoietic growth factors. In *Experimental Hematology Today, 1988*, Baum, S.J., Dicke, K.A., Lotzova, E.L. & Pluznik, D.H. (eds) p. 31. Springer-Verlag: New York.

HARIGAYA, K., CRONKITE, E.P., MILLER, M.E. & SHADDUK, R.K. (1981). Murine bone marrow cell line producing colony stimulating factor. *Proc. Natl Acad. Sci. USA*, **78**, 6963.

HARIGAYA, K. & HANDA, H. (1985). Generation of functional clonal cell lines from human marrow stroma. *Proc. Natl Acad. Sci. USA*, **82**, 3447.

HUNT, P., ROBERTSON, D., WEISS, D., RENNICK, L.F. & WITTE, O. (1987). A single bone marrow-derived stromal cell type supports the *in vitro* growth of early lymphoid and myeloid cells. *Cell*, **48**, 997.

KAWANO, F., YAMAGUCHI, K., NISHIMURA, H., TSUDA, H. & TAKATSUKI, K. (1985). Variation in the clinical course of adult T-cell leukemia. *Cancer*, **55**, 851.

OHKAWA, H., MIKATA, A., HARIGAYA, K. & UEDA, R. (1989). Novel functions and cellular interaction of human lymphoid stromal cells with lymphoid cell lines *in vitro*. *Exp. Hematol.*, **17**, 30.

RUNDLES, R.W. (1977). Chronic lymphocytic leukemia. In *Hematology*, Williams, W.J., Beutler, E., Erslev, A.J. & Rundles, R.W. (eds) p. 1002, McGraw-Hill: New York.

SCHRADER, J.W. & NOSSAL, G.J. (1980). Strategies for the analysis of accessary cell function: the *in vitro* cloning and characterization of the P cell. *Immunol. Rev.*, **53**, 61.

SNODGRASS, H.R., DEMBIC, Z.M., STEINMETZ, M. & BOCHMER, H.V. (1985). Expression of T cells antigen receptor genes during fetal development in the thymus. *Nature*, **315**, 232.

STEINMAN, R.M. & WITMER, M.D. (1978). Lymphoid dendritic cells are potent stimulators of the primary mixed leukocyte reaction in mice. *Proc. Natl Acad. Sci. USA.*, **75**, 5132.

TEW, J.G., THORBECKE, J.G. & STEINMAN, R.M. (1982). Dendritic cells in the immune response: characteristics and recommended nomenclature. *J. Reticuloendothel. Soc.*, **31**, 371.

TOROK-STORB, B. (1988). Cellular interactions. *Blood*, **72**, 373.

TSUDA, H. & TAKATSUKI, K. (1984). Specific decrease in T3 antigen density in adult T-cell leukemia cells: Flow microfluorometric analysis. *Br. J. Cancer*, **50**, 843.

WHITLOCK, C.A., TIDMARSH, G.F., MULLER-SIEBURG, C. & WEISSMAN, I.L. (1987). Bone marrow stromal cell line with lymphopoietic activity express high levels of a pre-B neoplasia-associated molecule. *Cell*, **48**, 1009.

ZIPORI, D. & SASSON, T. (1980). Adherent cells from mouse bone marrow inhibit the formation of colony stimulating factor (CSF) induced myeloid colonies. *Exp. Hematol.*, **8**, 816.